# FGF7-induced E11 facilitates cell-cell communication through connexin43

**DOI:** 10.7150/ijbs.65240

**Published:** 2021-09-03

**Authors:** Xiaoyu Liu, Mingru Bai, Yimin Sun, Xuchen Hu, Chenglin Wang, Jing Xie, Ling Ye

**Affiliations:** State Key Laboratory of Oral Diseases, National Clinical Research Center for Oral Diseases, West China Hospital of Stomatology, Sichuan University, Chengdu, 610041, China.

**Keywords:** Osteoblast, FGF7, E11, Gap Junction, Connexin43

## Abstract

Fibroblast growth factors (FGFs) include a large family of growth factors that play a critical role in maintaining bone homeostasis, but the specific role of its members such as FGF7 does not well understand. Osteoblasts are a kind of major cells essential for bone formation. Osteoblasts interact with one another to create the unique structure of osteons. The well-connected osteons constitute the cortical bone. As an early osteocyte marker that triggers actin cytoskeleton dynamics, E11 is essential for osteoblasts' dendrites formation. However, the upstream which regulates E11 is mainly unknown. The purpose of this study was to examine the influence of FGF7 on the expression and the distribution of E11 in osteoblasts, which mediated osteoblasts' processes formation and gap junctional intercellular communication (GJIC) partly through connexin43 (Cx43). We first demonstrated that FGF7 increased the expression of E11 in osteoblasts. We then showed that FGF7 promoted osteoblasts' dendrites elongation and functional gap junctions formation. Furthermore, E11 interacted directly with Cx43 in primary osteoblasts. MAPK pathway and PI3K-AKT pathway were involved in the effect of FGF7. Our results shed light on the unique role of FGF7 on osteoblasts, which may indicate that FGF7 plays a more significant role in the later stages of bone development and homeostasis.

## Introduction

The fibroblast growth factor (FGF) family comprises 22 members which are classified into canonical (secreted), endocrine, and intracellular members. The FGFs also can be grouped into seven subfamilies considering their biochemical functions, sequence similarities, and evolutionary relationships. Among five tyrosine kinases of FGFRs, FGFR1, FGFR2, and FGFR3 each have two alternative splicing variants with different ligand-binding specificity. In general, FGFRb splicing variants are found in epithelial tissues, and FGFRc splicing variants are found in mesenchyme tissues. It is well known that FGF/FGFR signaling plays an essential role in osteogenesis, while the effects depend on the type of FGFs and FGFRs expressed, the stage of cell maturation, and the microenvironment that may enhance or attenuate FGF/FGFR signaling in bone lineage cells 1, 2. As early as the end of the 20^th^ century, FGF7, also known as keratinocyte growth factor, is demonstrated to be a specific mitogen for epithelial cells 3. It is one of the paracrine and/or autocrine members of the FGF7 subfamily, including FGF3, FGF10, and FGF22 4. The only identified receptor for FGF7 is an alternative splicing variant FGFR2IIIb 5. Both *Fgf7* and *Fgfr2b* transcripts are found in the perichondrium and cartilage of limbs, ribs, vertebrae, pelvic bones, larynx, and trachea 6. Exogenous FGF7 facilitates osteogenic differentiation but not proliferation in mouse embryonic stem cells (ESCs) which is almost entirely attenuated by pretreating with anti-FGF7 antibody. The activation is partly mediated with ERK/Runx2 signaling pathway 7. Recombinant FGF7 improves bone formation of rat mandible defects and upregulates osteogenic markers, such as *Runx2*, *Sp7*, *Col-I*, *Sppl*, *Bglap*, and *Bmp2*. Similarly, FGF7 facilitates mineralization in rat bone marrow stromal cells (BMSCs) mediates with ERK and JNK signaling pathway. The addition of FGF7 also promotes BMSCs migration and increases the expression of SDF-1 and CXCR4 8. Our previous data revealed that the transcripts of *Fgf7* were higher compared with other FGF family members in MLO-Y4 osteocytes cell line and mice bone tissue. Treatment of FGF7 promoted the elongation of osteocyte cell processes and the expression of connexin43 (Cx43) partly through beta-catenin transduction 9.

E11 (also known as gp38 and podoplanin) is highly expressed in several cell types of dendritic morphology, including podocytes, type II lung alveolar cells, and osteocytes in the process of embedding or have recently embedded 10. The molecule has a role in dendrite formation, as inhibition of E11 in MLO-Y4 cells blocks dendrite elongation in response to fluid flow shear stress 11. However, upstream events which regulate E11 are mainly unknown. Numerous dendritic processes form a functional network of osteocytes, in which gap junctions involve the exchange of signaling molecules between adjacent cells. Gap junctions comprise 21 different connexins in humans and 20 in rodents. Connexin43, the most abundant connexin family member in bone, is expressed at the tip of osteocytes dendritic processes and between these processes and osteoblasts. Six identical or different connexin proteins can form one homomeric or heteromeric connexon, and two identical or different connexons can form one homomeric or heteromeric channel 12, 13. Intercellular communication mediated by gap junctions is critical to multiple aspects of the cell life cycle ranging from cell growth, migration, cell survival, and differentiation 14, 15. Inhibition of GJIC in osteoblasts delays their ability to differentiate and form mineralized matrix through a negative effect on the transcription of osteoblastic genes like *Alpl*, *Col1*, *Ibsp*, and *Bglap* 16-18. In particular, Cx43 is vital for animal embryogenesis and bone development. Mice with *Cx43* global deletion died immediately after birth due to neural crest cell defects and osteoblast dysfunctions 19. Conditional deletion of *Cx43* in osteoblasts and osteocytes were viable but exhibited osteopenia 20-22. In the past decades, it has become clear that gap junctions have been made up not only of pore-forming connexins but also of several other proteins. Giepmans BN et al. demonstrated that Cx43 interacts with the second PDZ domain of the zona occludens-1 protein 23. Yang et al. confirmed that N-cadherin interacts with Cx43 at the site of cell-cell contact and knockdown of N-cadherin decreases the TGF-β1-induced cell-cell communication via Cx43 24. Kanemitsu MY et al. showed that the proline-rich region of Cx43 binds to the Src homology 3 (SH3) domain of v-Src and Tyrosine-phosphorylated Cx43 forms a docking site for the Src homology 2 (SH2) domain of v-Src 25.

However, the underlying mechanisms remain unclear as the interaction between cellular proteins and connexins has not yet been fully determined and the biological events related to the regulation of osteoblasts through FGF7 are largely unspecified. In this study, we demonstrated that FGF7 increased the expression of E11 and Cx43 and elongated the osteoblast processes. Moreover, we demonstrated a direct interaction between E11 and Cx43 and FGF7-induced cell-cell communication required E11 in primary osteoblasts. Last but not the least, MAPK pathway and PI3K-AKT pathway were involved in the effect of FGF7. This paper may serve as a new mechanism for the understanding of gap junctions function and FGF7 signaling.

## Materials and methods

### Cell culture

The materials used for this study were obtained according to ethical principles, and the protocol was reviewed and approved by our institutional review board (Institutional Review Board at the West China Hospital of Stomatology, No. WCHSIRB-D-2017-029). Osteoblasts were isolated from the calvaria of 1-day-old mice. Briefly, the mouse calvaria was collected with ophthalmic scissors. The epidermis of the calvaria was stripped. The collected calvaria was cut into small pieces and digested by trypsin (0.25%) for 30 min. The trypsin contained supernatant was then removed and replaced with 0.1% collagenase type I for 12 h. The collagenase type I-treated solution (osteoblasts suspension) was collected and mixed 1:1 (V/V) with fresh 10% heat-activated fetal bovine serum (FBS), α-MEM. The mixed suspension was centrifuged at 1000 r/min for 5 min. After removing the supernatant, the 10% FBS α-MEM was added into a centrifuge tube to resuspend the osteoblasts. Then, the suspended osteoblasts were seeded into 25 cm^2^ flasks at 37 °C in a humidified atmosphere of 5% CO2 till usage. MC3T3-E1 cell line were cultured in a 25 cm^2^ flask containing 3-4 ml α-MEM with 10% fetal bovine serum at 37 °C in a humidified atmosphere of 5% CO_2_ in the air.

### FGF7 treatment

Both primary osteoblasts and MC3T3-E1 cell line were seeded onto six-well plates at 5 × 10^5^ cells per well (85%~95% confluence). Osteoblasts were allowed to equilibrate for 12 h. Culture media were then replaced with 2% FBS α-MEM for a 12 h starvation. Then, osteoblasts were divided into two groups. In the treatment group, the medium was replaced with fresh 1% FBS α-MEM containing 10 ng/ml FGF7 (Recombinant Mouse KGF/FGF7, R&D Systems, Minnesota, USA, 5028-KG-025/CF). At the protein level, cell lysate samples were collected at 24 h for western blot.

### RNA sequencing

The primary osteoblasts were cultured onto six-well plates at 5 × 10^5^ cells per well (85%~95% confluence) for 12 h and followed starvation for 12 h in 2% FBS α-MEM. Then, osteoblasts were divided into two groups. In the treatment group, the medium was replaced with fresh 1% FBS α-MEM containing 20 ng/ml FGF7 for 24 h. Cell lysate samples were collected using Trizol (No. 15596-026, Thermo Fisher Scientific). Three independent repeats based on different primary osteoblasts were carried out. The cell samples were sent for transcriptome analysis at Shanghai Lifegenes Biotechnology CO., Ltd (Shanghai, China). Before transcriptome sequencing, the RNA integrity was assessed using the RNA Nano 6000 Assay Kit of the Bioanalyzer 2100 system (Agilent Technologies, CA, USA). According to the manufacturer's instruction, a total amount of 1.5 μg RNA per sample was used as the input material for the RNA sample detection. The clustering of the index-coded samples was performed on a cBot Cluster Generation System using HiSeq 4000 PE Cluster Kit (Il-Lumia, San Diego, CA, USA). Raw data (raw reads) of fast q format were firstly processed through in-house Perl scripts. In this step, clean data (clean reads) were obtained by removing reads containing adapter, reads containing ploy-N, and low-quality reads from raw data. Paired-end clean reads were aligned to the reference genome using HISAT2 v2.1.0. In data analysis, HTSeq v0.6.1 was used to count the reads numbers mapped to each gene. Gene FPKMs were computed by summing the FPKMs of transcripts in each gene group.

### Immunohistochemical staining

Paraffin sections were dewaxed in xylene, rehydrated with distilled water, and then subjected to antigen retrieval. Tissue sections were washed using PBS with 0.25% Triton-X100 and stained based on the standard protocol for the rabbit HRP-DAB Kit. Fast green and hematoxylin were used to counterstain the background, and a neutral balsam was used as a mounting medium. A microscope imaging system (Olympus BX53) was used for imaging. The primary antibodies involved were FGF7-antibody (1:200) (Thermo, Waltham, USA, PA5-49715).

### Immunofluorescence staining

Osteoblasts were seeded onto a 35 mm Glass Bottom Dish and allowed to equilibrate for 12 h. Osteoblasts were then cultured for 24 h with fresh 10% FBS α-MEM containing either 10 ng/ml FGF7 or PBS solution. After the treatments, the cells were washed three times for 2 min and fixed with 4% paraformaldehyde for 10 min, followed by three rinsed with PBS. Then they were permeabilized with 0.25% Triton X-100 for 5 min, washed with PBS. Afterward, the cells were blocked with 5% bovine serum albumin for 60 min, washed with PBS, followed by the addition of E11 antibody (1:200) (R&D Systems, AF3244) and connexin43 antibody (1:200) (Abcam, ab11370) and then incubated overnight at 4 °C. Following incubation, the cells were washed three times for 15 min with PBS. Either Donkey anti-goat IgG (Abcam, ab150135) or Donkey anti-rabbit IgG (Abcam, ab150075) was added and incubated for 2h at room temperature. Then, FITC-conjugated phalloidin (Invitrogen, CA, USA) in PBS was added for double staining and incubated overnight at 4 °C. Followed incubation, the cells were washed three times for 5 min and then stained by DAPI (Sigma, Aldrich, Louis, MO, USA) for nucleus staining. The cell images were captured using a modified confocal laser scanning microscope. (A1R MP+, Nikon, Tokyo, Japan and Olympus, FV3000, Japan) and analyzed with Image-Pro Plus Software 6.0.

### Western blotting

Protein samples were prepared by mixing one part of the sample with one part of sample buffer and then boiled at 100 °C for 5 min. Proteins were separated in 8-12% sodium dodecyl sulfate-polyacrylamide gel electrophoresis and transferred to a polyvinylidene difluoride membrane at 200 mA for 2 h in ice treatment. The blot was blocked with 5% fat-free dry milk suspended in 1X TBST for 1 h at room temperature. The resulting blot was incubated with antibodies (1:1000-2000), including beta-actin (sc-47778, Santa Cruz Biotechnology, Cambridge, UK); E11; Cx43; ERK (343830, ZEN BIO, China); P-ERK (301245, ZEN BIO, China); SAPK/JNK (380556, ZEN BIO, China); Phospho-SAPK/JNK (Thr183, 381100, ZEN BIO, China); P38 (340697, ZEN BIO, China); P38-MAPK (Phospho-Thr180/Tyr182, 310091, ZEN BIO, China); Akt (342529, ZEN BIO, China); p-Akt (Phospho-Ser473, 310021, ZEN BIO, China) for overnight at 4 °C, followed by incubation with 1:5000 anti-IgG-HRP for 2 h at room temperature. Signals from the blots were obtained using a Western Blotting Luminol Reagent Kit.

### Scrape loading and dye transfer assay

The scrape loading/dye transfer (SL/DT) technique, which relies on the introduction of small molecular (MW < 900) dyes (Lucifer Yellow, MW457, L0259, sigma) and tracing their intercellular movement through gap junctions, is applied to assess the effect of FGF7 on the GJIC between osteoblasts. Lucifer Yellow cannot get into intact cells unless the cell membranes are torn transiently produced by scrape loading. Both primary osteoblasts and MC3T3-E1 cell line were treated with FGF7 for 24 h, then were rinsed with Ca Mg-PBS and scraped by a surgical blade before the addition of fluorescent dye (1 mg/ml Lucifer Yellow). After a 5-10 min incubation at room temperature, the cells were washed thoroughly with PBS and fixed by adding 4% paraformaldehyde. The cells were imaged with CLSM (Nikon&Olympus).

### Co-Immunoprecipitation

Protein immunoprecipitation was performed using the Pierce® Co-immunoprecipitation Kit (Lot#: SB240573B, Thermo Fisher Scientific, Waltham, MA). Briefly, primary osteoblasts were lysed according to the manufacturer's instructions, and the antibody of the bait protein was added to the lysate at a 1:30 ratio. After overnight incubation, the antigen (bait protein) and the interacting proteins (prey proteins) were separated and purified with a centrifugal column provided in the kit. After collection with elution buffer, the samples were blotted onto the PVDF membrane via western blot.

### Small interfering RNA transfection

Cells at 70%-80% confluence were transfected with E11 small interfering RNA (siRNA) or negative control siRNA. Lipofectamine RNAiMAX reagent (Invitrogen) was used according to the manufacturer's instructions. The proteins were collected after transfection for 24 hours.

### Quantitative real-time PCR (QPCR)

Extraction and purification of the total RNA were conducted using the RNeasy Plus Mini Kit (Qiagen, CA, USA). Trizol reagent was firstly added, then the subsequent procedures were carried on according to the manufacturer's instructions. After quantification using the NanoDrop® spectrophotometer (Nanodrop Spectrophotometer 2000c, Thermo Scientific, Waltham, USA), purified RNA samples were used to synthesize cDNA reverse transcription with the reverse transcript kit (Thermo Scientific, Vilnius, Lithuania). According to the manufacturer's protocols, qPCR was performed with SYBR Premix Ex Taq (Takara, Tokyo, Japan) in a qPCR system (Applied Biosystems, Foster City, CA, USA). PCR reactions were conducted at 0.5 μM for each primer in a 25 μl volume containing 1 μl cDNA. The reaction was initiated by activating the polymerase with a 5 min pre-incubation at 95 °C. Amplification was achieved with 45 cycles of 15-sec denaturation at 94 °C, 20-sec annealing at 65 °C, and 10-sec extension at 72 °C. The melting curve analysis concluded the program. Each sample was performed in triplicate. The fold change of each gene was determined by the 2-∆∆Ct method. GAPDH was used as an internal control to normalize the data.

### Statistical analysis

All experiments were performed in triplicate and reproduced at least three separate times. Statistical analysis of the data was performed with SPSS 16.0 using one-way analysis of variance (ANOVA) or t-test to determine whether differences existed. The data were expressed as means ± standard deviation (SD). The critical significance level was set to p < 0.05 (*).

## Results

### The high expression of *Fgf7* among all FGF ligand members of osteoblasts and the distribution of FGF7 in bone tissue

**Figure 1:**
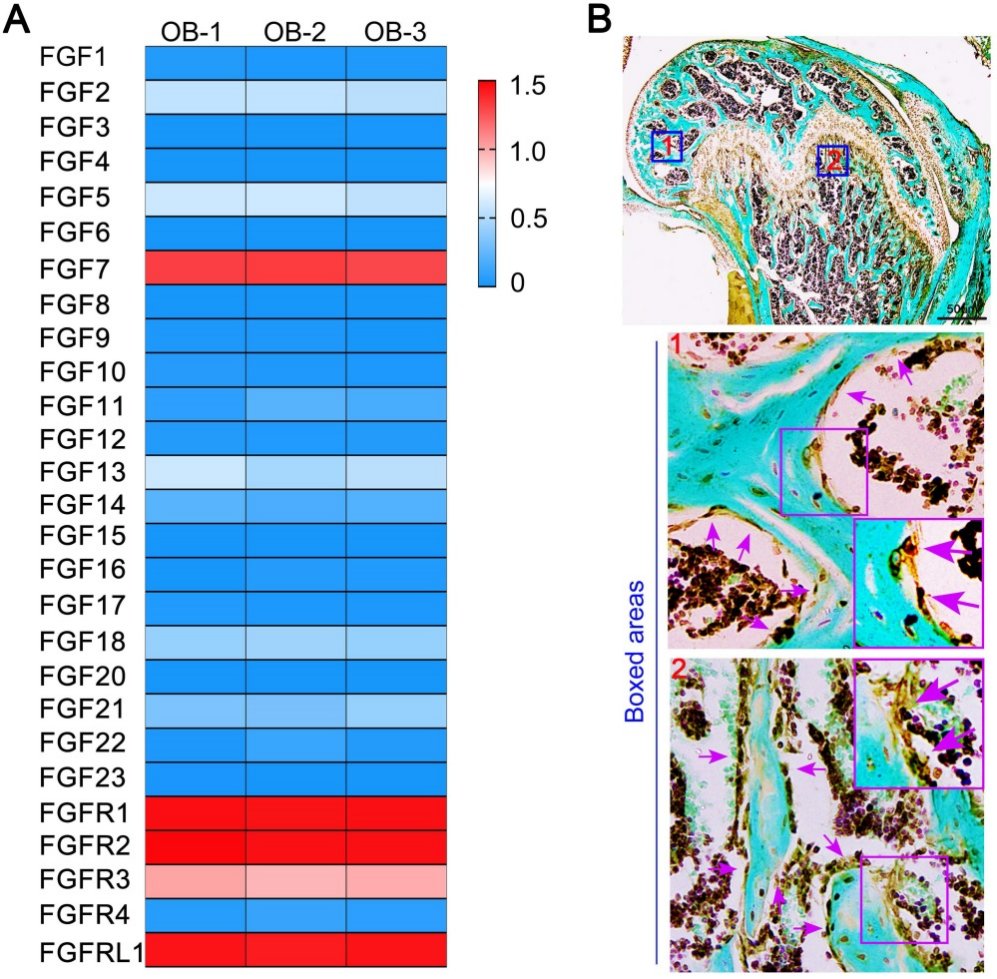
** The high expression of *Fgf7* of osteoblasts and the distribution of FGF7 in bone tissue. (A)** RNA sequencing results showing the whole fibroblast growth factor family members and receptor members of primary osteoblasts. The data were presented as log (FPKM + 1) and showed the higher mRNA expression of *Fgf7* among other FGF ligand members. FPKM: Fragments per Kilobase of transcript per Million fragments mapped. The results were based on three independent samples (n = 3). **(B)** IHC showing the distribution of FGF7 in the femur of 4 weeks wild-type C57 mice. Fast green is used to counterstain the background. Boxed areas further showing that FGF7 is partly generated by osteoblasts on the surface of trabecular bone within the secondary ossification center (boxed area 1) and subchondral bone (boxed area 2).

After analysis of RNA sequencing, we identified the expression of the whole fibroblast growth family members and receptor members of primary osteoblasts. We found that the expression of *Fgf7* was more remarkable than other ligand members (Fig. 1A). We next detected the expression of the intact fibroblast growth family members in primary osteoblasts and MC3T3-E1 cell line by qPCR. We found that the expression of *Fgf7* was higher than other ligand members in both two cells (Fig. S1A & S1C). Our data also confirmed that FGFR2IIIb, as the only identified receptor for FGF7 is present in both primary osteoblasts and MC3T3-E1 cell line (Fig. S1B & S1D). The high expression of *Fgf7* prompted us to test its biological function of osteoblasts further. We first detected the distribution of FGF7 in the femur of 4 weeks wild-type C57 mice. Our results verified that FGF7 was partly generated by osteoblasts on the surface of trabecular bone within the secondary ossification center and subchondral bone by IHC (Fig. 1B). The FGF7 antibody also stained the periosteum, the perichondrium, the bone marrow, the entire growth plate, and the osteocytes embedded in the osseous lacuna.

### FGF7 increases the expression of E11 in osteoblast lineage

**Figure 2:**
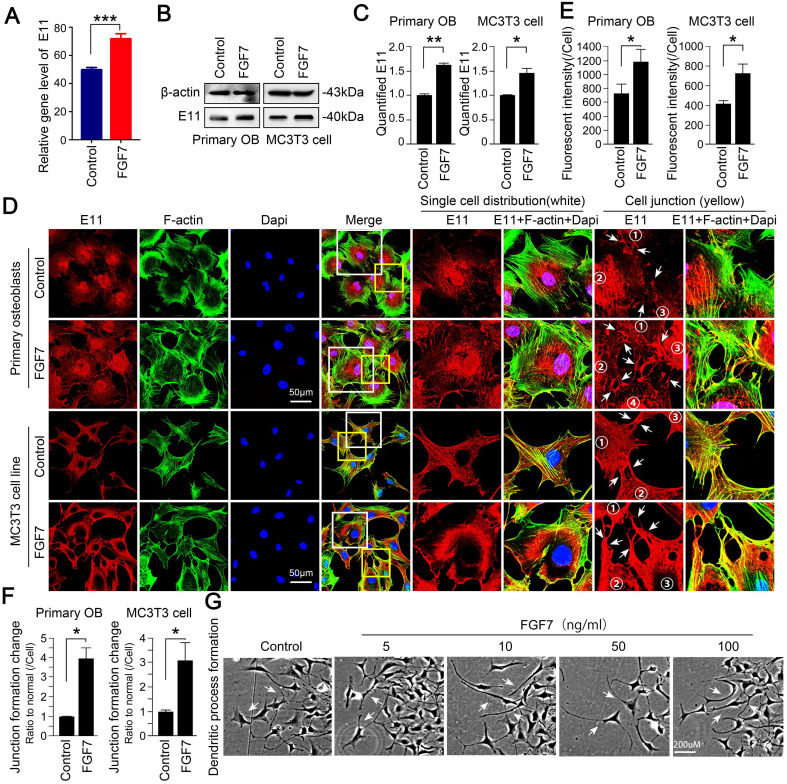
** FGF7 promotes the expression of E11 in primary osteoblasts and MC3T3-E1 cell line. (A)** RNA sequencing results showing the changes of *E11* in response to FGF7 in 24 h. The data were presented as the ratio of FPKM to the inner beta-actin levels. FPKM: Fragments per Kilobase of transcript per Million fragments mapped. Cell samples from each group were obtained from three independent cell isolates (n = 3). **(B)** Western blotting showing the protein expression of E11 in primary osteoblasts and MC3T3-E1 cell line after treatment of FGF7 (10 ng/ml) for 24 h. beta-actin was used as the loading control. **(C)** Quantification of western blot analysis in (B). The results were based on three independent experiments (n = 3). * p < 0.05, ** p < 0.01. **(D)** Representative IF staining by CLSM showing the increased expression of E11 in primary osteoblasts and MC3T3-E1 cell line in response to FGF7 (10 ng/ml). F-actin (green) and Dapi (blue) were used to counterstain the background. The white boxed areas are showing the distribution of E11 in single cells. The yellow boxed areas are showing the distribution of E11 at cell junctions. The white arrows are revealing the details of the distribution of E11. **(E)** Quantification of E11 fluorescent intensity in (D) by Image J. The results were based on three independent experiments (n = 3). * p < 0.05. **(F)** Quantification further showing the changes in cell junction numbers induced by FGF7. The results were based on three independent experiments (n = 3). * p < 0.05. **(G)** Bright field showing the cell morphology at the edge of the scratch wound after FGF7 induction in a concentration-dependent manner for 36 h in MC3T3-E1 cell line and the white arrows showing the elongated osteoblasts cell processes.

After analysis of RNA sequencing, we found the increased changes of *E11* in primary osteoblasts in response to FGF7 (Fig. 2A). We next performed Western blotting assay to examine the effect of FGF7 on E11 protein expression and found that the expression of E11 increased in response to FGF7 (10 ng/ml) in two kinds of osteoblasts (Fig. 2B). The expression of E11 was up to 1.60-fold and 1.45-fold compared with control after quantification, respectively (Fig. 2C). Next, to study the distribution of E11 in osteoblasts induced by FGF7, cell immunofluorescence staining was performed, and we further confirmed that FGF7 could modulate the expression and the distribution of E11 (Fig. 2D). Cells treated with FGF7 (10 ng/ml) for 24 hours exhibited a dendritic shape characteristic of osteocytes (white boxed area in the middle column). They displayed numerous cell processes to connect with adjacent cells (yellow boxed area in the right column). The distribution of E11 seemed more toward the cell membrane in both primary osteoblasts and MC3T3-E1 cell line after FGF7 treatment. Fluorescence OD measurement quantified the changes in two kinds of cells (Fig. 2E). The quantification of cell junctions formation further upheld the results (Fig. 2F). We further found that osteoblasts treated with FGF7 extended long processes to connect to neighboring cells and formed intercellular communications in a concentration-dependent manner (Fig. 2G).

### FGF7 promotes gap junction intercellular communication in osteoblast lineage

**Figure 3:**
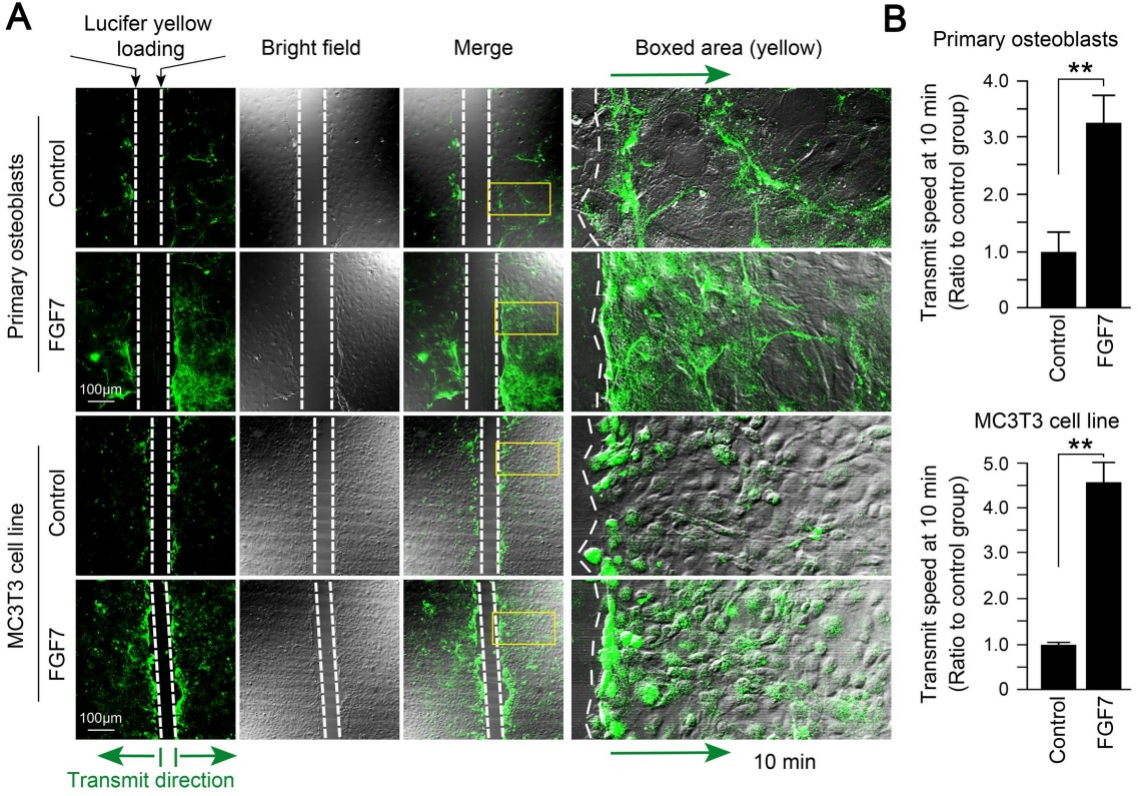
** FGF7 promotes gap junction intercellular communication in primary osteoblasts and MC3T3-E1 cell line. (A)** The scrape loading/dye transfer (SL/DT) assay showing functional GJIC in living primary osteoblasts and MC3T3-E1 cell line treated with FGF7 (10 ng/ml). The Lucifer yellow dye enters cells at the scratch (dotted white lines) and is transferred to cells distant from the scratch (green arrows). Intercellular gap junction transfer was calculated by measuring the distance from the scratching edge to the most distant cells with Lucifer yellow uptake. The boxed area further showing the different transmission speeds in response to FGF7. **(B)** Quantification showing different transmission speeds of Lucifer yellow in response to FGF7. The results were based on three independent experiments (n = 3). ** p < 0.01.

With the scrape loading/dye transfer (SL/DT) assay, we found that the formation of gap junctions induced by FGF7 (10 ng/ml) was much more and showed higher transmission speed of fluorescent dyes compared to control in both primary osteoblasts and MC3T3-E1 cell line (Fig. 3A). The quantification showed that the 7.9-fold and 6.1-fold owned the transmission speed of Lucifer yellow dye in the FGF7 group compared to that in control (Fig. 3B).

### FGF7 upregulates the expression of connexin43 in osteoblast lineage

**Figure 4:**
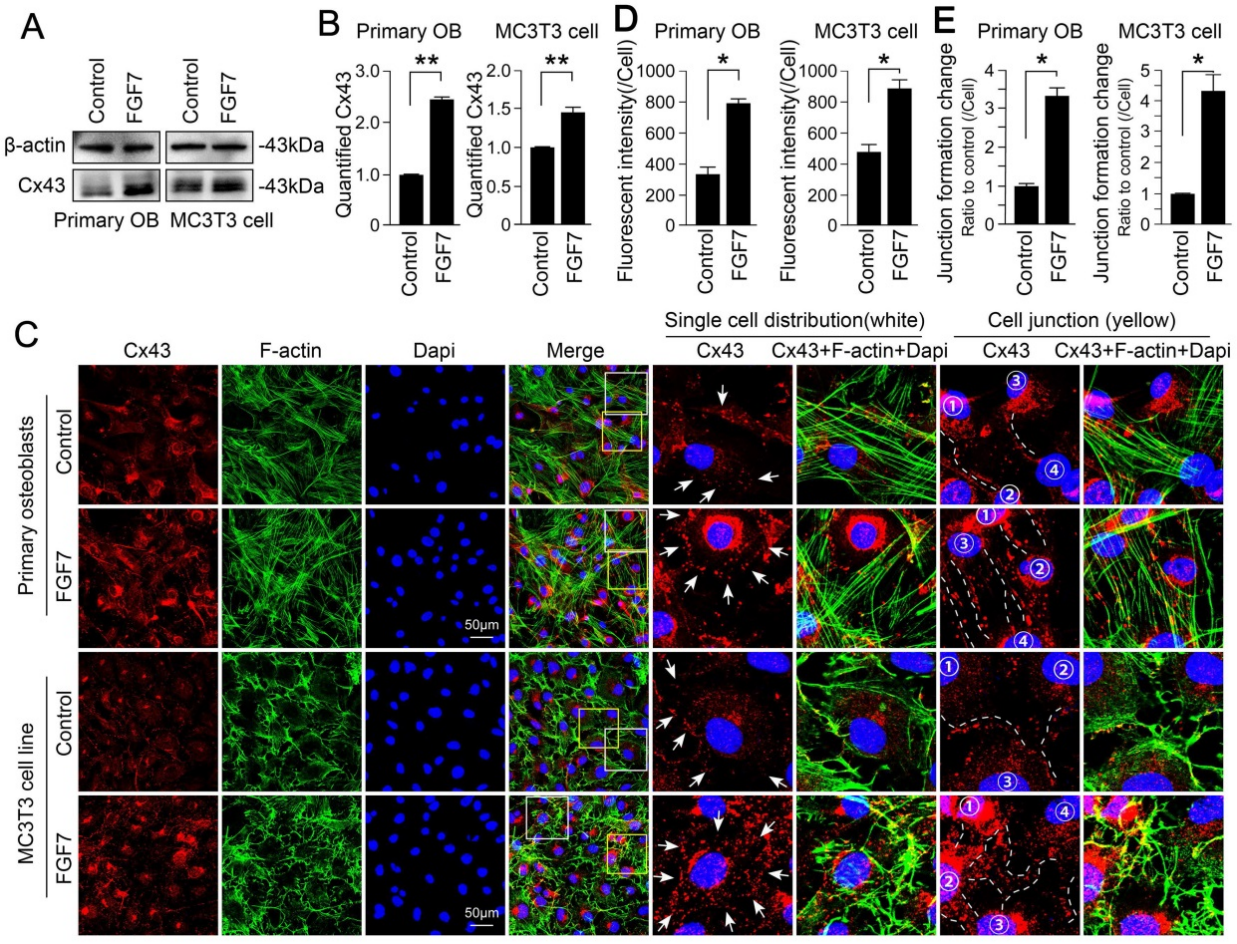
** FGF7 increases the expression of connexin 43 in primary osteoblasts and MC3T3-E1 cell line. (A)** Western blotting showing the protein expression of Cx43 in primary osteoblasts and MC3T3-E1 cell line after treatment of FGF7 (10 ng/ml) for 24 h. beta-actin was used as the loading control. **(B)** Quantification of western blot analysis in (A). The results were based on three independent experiments (n = 3). ** p < 0.01. **(C)** Representative IF staining by CLSM showing the increased expression of Cx43 in primary osteoblasts and MC3T3-E1 cell line in response to FGF7 (10 ng/ml). F-actin (green) and Dapi (blue) were used to counterstain the background. The white boxed areas showing the distribution of Cx43 in single cells. The yellow boxed areas showing the distribution of Cx43 in cell junctions. The white arrows and dotted lines showing the details of the distribution of Cx43. **(D)** Quantification of Cx43 fluorescent intensity in (C) by Image J. The results were based on three independent experiments (n = 3). * p < 0.05. **(E)** Quantification further showing the changes in cell junction numbers induced by FGF7. The results were based on three independent experiments (n = 3). * p < 0.05.

Western blot assay effectively determined that the up-regulation of Cx43 in response to FGF7 (10 ng/ml) in two kinds of osteoblasts (Fig. 4A). The increase in response to FGF7 was up to 2.46-fold and 1.45-fold compared with control groups after quantification, respectively (Fig. 4B). To continue to study the distribution of Cx43 in osteoblasts treated with FGF7, we performed cell immunofluorescence staining and found that Cx43 exhibited spot-like expression and distributed along with the osteoblast cell processes, even at the sites of cell-cell contact (Fig. 4C and white boxed area in the middle column). Besides, in an analysis of cell junctions, we found that cells treated with FGF7 had more Cx43 localized along with the cytoskeleton (yellow boxed area in the right column). Fluorescence OD measurement quantified the changes in two kinds of cells (Fig. 4D). The quantification of cell junctions formation further confirmed the results (Fig. 4E).

### Connexin43 and E11 has a direct interaction

**Figure 5:**
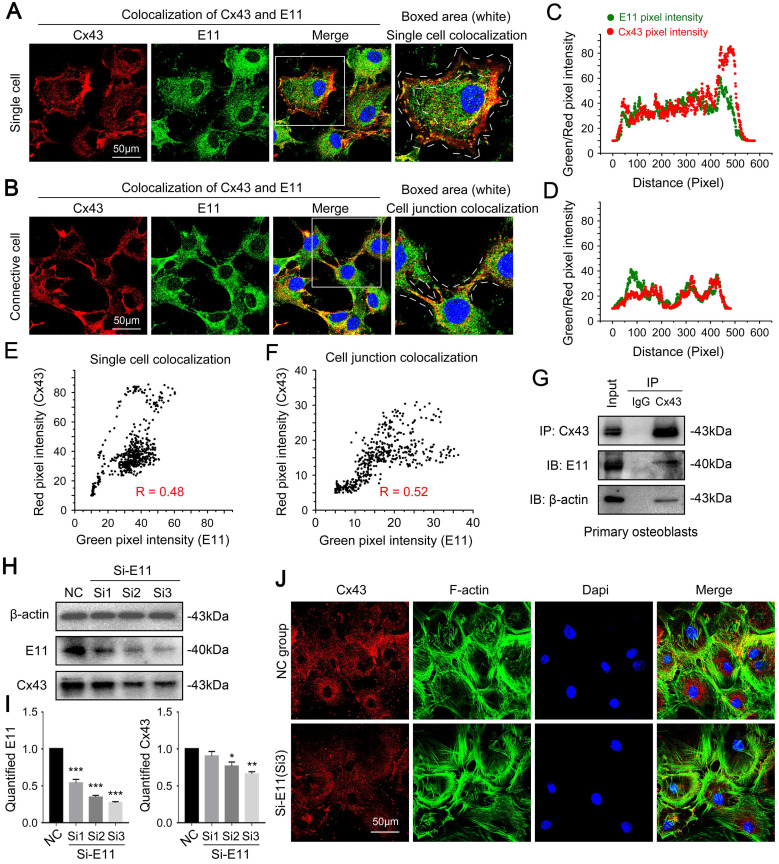
** Connexin 43 interacts with E11 in osteoblasts. (A)** Double immunofluorescent staining showing colocalization of Cx43 (red) and E11 (green) proteins in primary single osteoblasts. Boxed areas are showing the details of the colocalization of two proteins in single cells. **(B)** Double immunofluorescent staining showing colocalization of Cx43 (red) and E11 (green) proteins in connective primary osteoblasts. Boxed areas are showing the details of colocalization of two proteins in cell junctions. **(C, D)** Intensity traces by Image J showing the colocalization distributions of Cx43 (red curves) and E11 (green curves) corresponding to (A) and (B). The results were based on three independent experiments (n = 3). **(E, F)** Scatter grams showing the Pearson's correlation of Cx43 and E11 in cellular boundary colocalization corresponding to (A) and (B). The results were based on three independent experiments (n = 3). **(G)** Co-IP assay further showing the interaction between Cx43 and E11. Cx43 antibody was used as bait protein to prey E11 and beta-actin. The IgG (rabbit) group was used as the negative control. The results were based on three independent experiments (n = 3). **(H)** Western blotting showing the protein expression of Cx43 in response to three E11 small interfering RNA named Si1, Si2, Si3 in primary osteoblasts. **(I)** Quantification of western blot analysis in (H). The results were based on three independent experiments (n = 3). * p < 0.05, ** p < 0.01, *** p < 0.001. **(J)** Representative IF staining by CLSM showing the distribution of Cx43 in primary osteoblasts after treatment of E11 small interfering RNA named Si3.

Next, we explored the potential mechanism founded on the observed phenomenon. We found coincident changes with the expression and the distribution between Cx43 and E11 by co-immunofluorescent staining analysis on CLSM in single cells (Fig. 5A) and connective cells (Fig. 5B). Boxed areas showed the details of the colocalization of Cx43 and E11. Fluorescence intensity profiles of Cx43 (red curves in Fig. 5C and 5D) and E11 (green curves in Fig. 5C and 5D) are indicated, respectively. The scatter diagram together with Pearson's correlation coefficients for the overlap between red (Cx43) and green (E11) pixel intensities correspond to the single-cell (R = 0.48) (Fig. 5E) and the cell junctions (R = 0.52) (Fig. 5F). Interaction between Cx43 and E11 was further confirmed through co-immunoprecipitation assay (Fig. 5G). The results also showed that Cx43 had an indirect interaction with beta-actin, which is the microfilament correlate with the cell cytoskeleton. To further prove the crosstalk between Cx43 and E11, we used small interfering RNA to knock down the expression of E11 in primary osteoblasts. It was found that the inhibition of E11 decreased the protein level of Cx43 (Fig. 5H). The quantification further upheld the results (Fig. 5I). Moreover, the inhibition of E11 reduced the expression of Cx43, which was observed directly by cell immunofluorescence (Fig. 5J).

### FGF7-induced cell-cell communication through Cx43 requires E11

**Figure 6:**
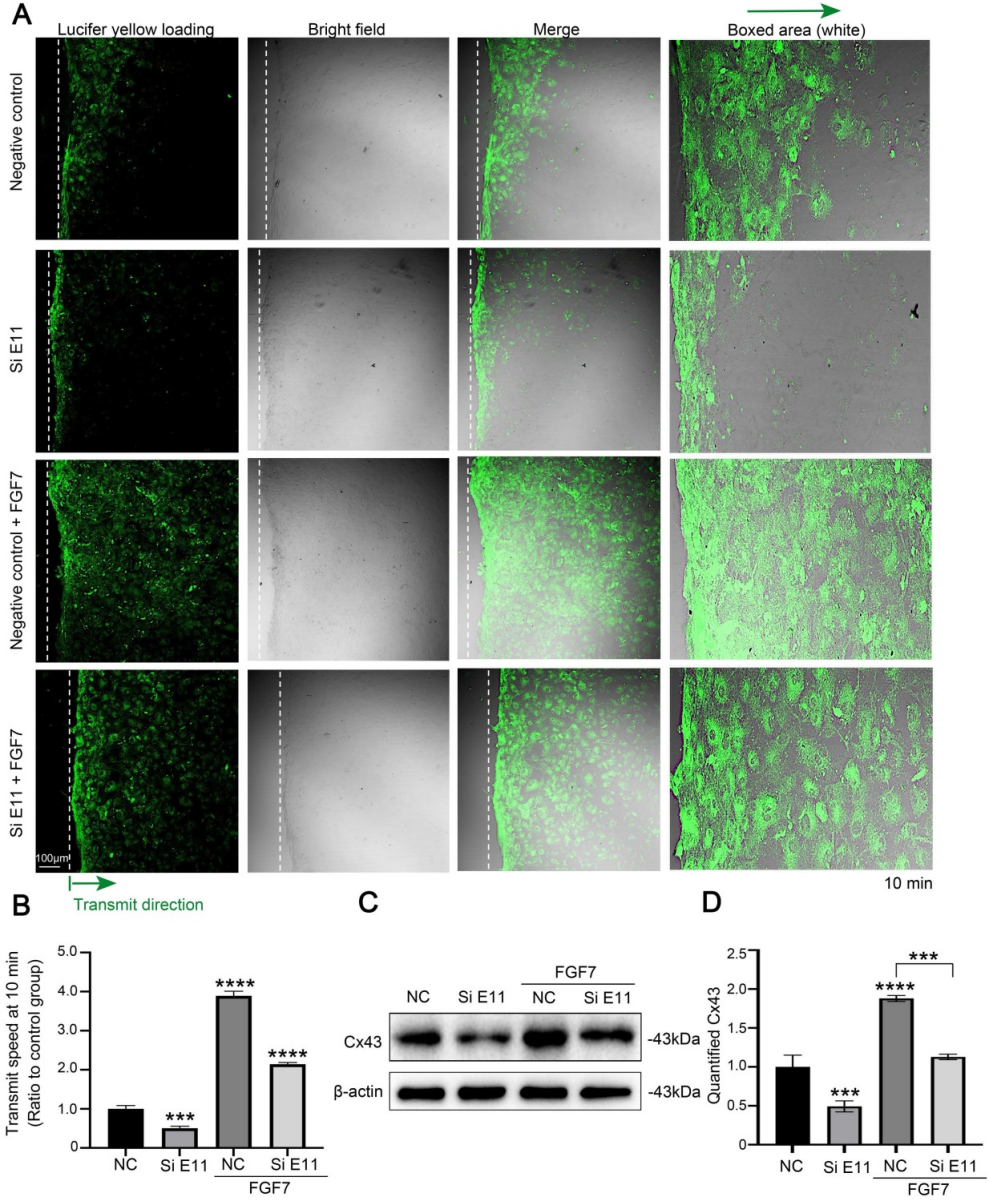
**E11 is involved in the FGF7-induced functional GJIC and up-regulation of Cx43. (A)** The scrape loading/dye transfer (SL/DT) assay showing functional GJIC in living primary osteoblasts treated with FGF7 (10 ng/ml) in the presence or absence of E11 siRNA. The Lucifer yellow dye enters cells at the scratch (dotted white lines) and is transferred to cells distant from the scratch (green arrows). Intercellular gap junction transfer was calculated by measuring the distance from the scratching edge to the most distant cells with Lucifer yellow uptake. The boxed area further showing the different transmission speeds. **(B)** Quantification showing different transmission speeds of Lucifer yellow in (A). The results were based on three independent experiments (n = 3). *** p < 0.001, **** p < 0.0001. **(C)** Western blotting showing the protein expression of Cx43 in primary osteoblasts treated with FGF7 (10 ng/ml) in the presence or absence of E11 siRNA. **(D)** Quantification of western blot analysis in (C). The results were based on three independent experiments (n = 3). ***p < 0.001, **** p < 0.0001.

To determine the role of E11 for the FGF7-induced GJIC activity through Cx43, we performed scrape loading/dye transfer (SL/DT) assay. The results showed that FGF7 treatment increased GJIC activity, whereas knockdown of E11 attenuated the GJIC activity and also weakened the FGF7-induced increase in GJIC activity (Fig. 6A). The quantification of the transmission speed of Lucifer yellow dye further confirmed the results (Fig. 6B). Western blotting assay showed that knockdown of E11 also attenuated the stimulatory effect of FGF7 on Cx43 protein level (Fig. 6C). The quantification further upheld the results (Fig. 6D).

### MAPK signaling pathway and PI3K-AKT signaling pathway are involved in the role of FGF7 induced E11 and Cx43

**Figure 7:**
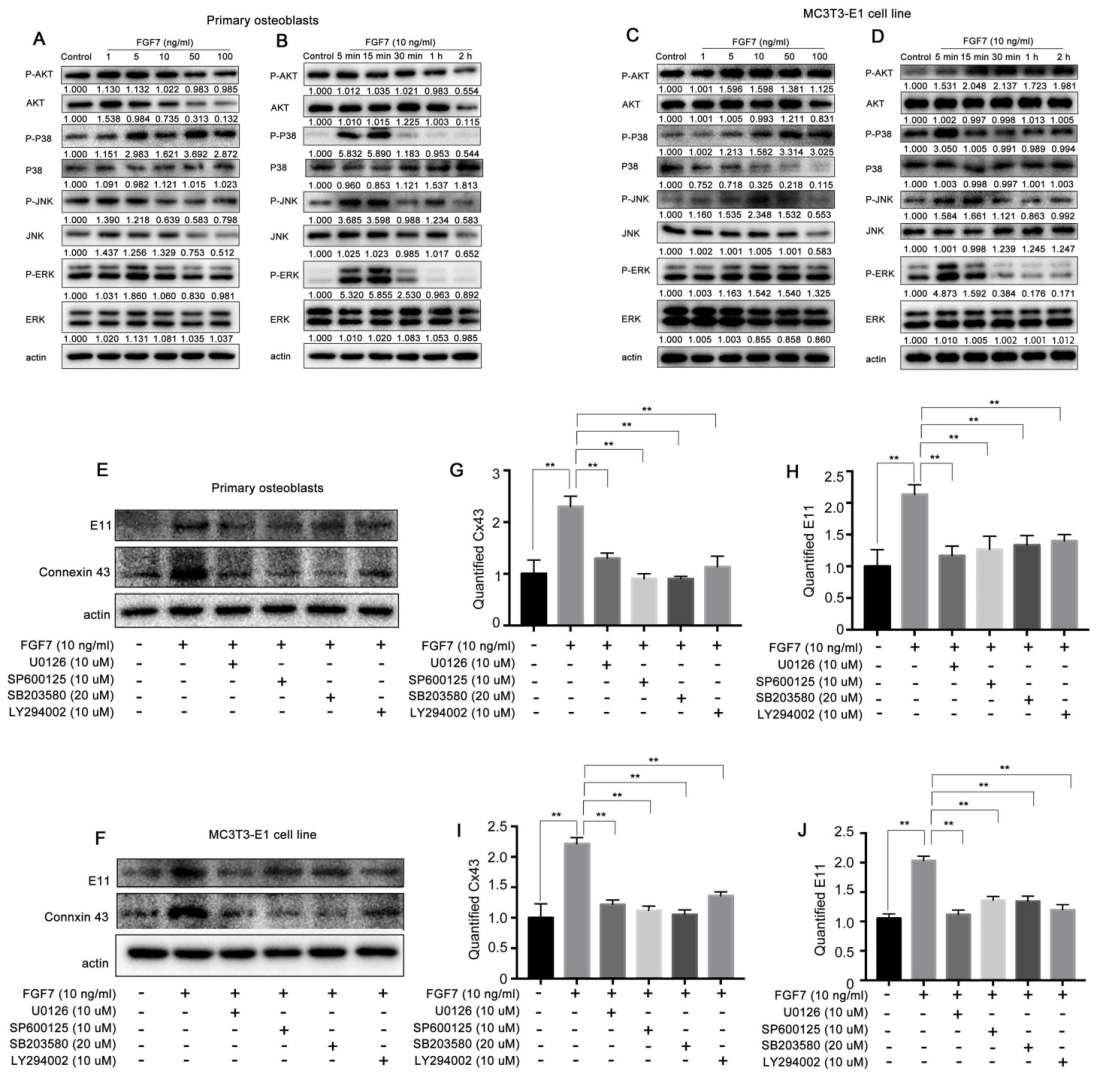
** MAPK pathway and PI3K-AKT pathway are involved in the regulation of E11 and Cx43 in response to FGF7. (A, C)** Western blotting showing ratios of P-ERK/ERK, P-JNK/JNK, P-P38/P38, and P-AKT/AKT increased in a concentration-dependent manner in primary osteoblasts and MC3T3-E1 cell line after treatment of FGF7 for 2 h. beta-actin was used as the loading control. The results were based on three independent experiments (n = 3). **(B, D)** Western blotting showing ratios of P-ERK/ERK, P-JNK/JNK, P-P38/P38, and P-AKT/AKT increased in a time-dependent manner in primary osteoblasts and MC3T3-E1 cell line after treatment of FGF7 (10 ng/ml) within 2 h. beta-actin was used as the loading control. The results were based on three independent experiments (n = 3). **(E, F)** Western blotting showing that the specific inhibitors attenuated the stimulation effect of FGF7 at E11 and Cx43 expression in primary osteoblasts and MC3T3-E1 cell line. **(G-J)** Quantification of western blot analysis in (E, F). The results were based on three independent experiments (n = 3). ** p < 0.01.

Canonical FGF signaling activates the MAPK, PI3K-AKT, PLC/PKC pathways. To determine the signaling pathway mediated with FGF7 in osteoblasts, Western blot assay was performed. It was found that the protein levels of P-ERK, P-JNK, P-P38, and P-AKT were increased within 2 h post-FGF7 treatment of two kinds of osteoblasts in both concentration-dependent and time-dependent manner, especially the increased levels of P-ERK and P-P38 were more significant than other signaling pathways (Fig. 7A-D). There have no obvious effect on P-PLCG and P-PKC protein levels in response to FGF7 (Fig. S2A, S2B). We then tested if the MAPK and PI3K-AKT signaling pathways were associated with the expression of E11 and Cx43 by applying specific inhibitors (U0126 ERK inhibitor, SP600125 JNK inhibitor, SB203580 P38 inhibitor, and LY294002 AKT inhibitor). Results showed that the specific inhibitors attenuated the stimulation effect of FGF7 at E11 and Cx43 production of two kinds of osteoblasts (Fig. 7E,F). The quantification further confirmed the results (Fig. 7G-J).

## Discussion

FGF7 is a paracrine/autocrine member of the FGF family and exclusively mediates biological functions by activating FGFR2b 5. The original importance of FGF7 lies in its role as a powerful mitogen in various epithelial cells 3, 26-28. Subsequent studies have demonstrated that FGF7 also exhibits other biological functions, such as regulating cell migration, differentiation, preventing apoptosis, promoting branching morphogenesis, and cytoprotection 29. Although *Fgf7* knockout mice appeared normal and only revealed subtle phenotypes involving the hair, kidneys, and the bladder, later studies suggest that FGF7 seem to have played a more significant role in the later stages of tissue development and homeostasis 29. However, the importance of FGF7 in bone biology is appearing in recent years. The functions of FGF7 in bone formation, bone diseases, and downstream signaling are still lacking.

Of the most abundant cells in bone, osteocytes are terminally differentiated osteoblasts derived from mesenchymal stem cells (MSCs) and reside in lacunae with their long dendritic processes to maintain communications with others. Instead of being a passive cell, it is now clear that osteocytes play a critical role in bone homeostasis in the adult skeleton 30-32. While signaling that direct differentiation from MSCs to osteoblasts has been extensively studied, the mechanism during the transition from the cuboidal-like osteoblasts to dendritic-like osteocytes is just beginning to be elucidated. The changes in cytoskeleton proteins, hormones, unique markers, and the upstream factors during this transition remain scarce 10, 33. As osteocytes can survive for up to decades and are involved in many critical physiological functions, such as maintaining mineral metabolism, acting as mechanosensors, and secreting endocrine hormones to communicate with other organs, it is necessary to understand the factors that influence osteocytes differentiation 34.

The osteoblast-to-osteocyte transition is often referred to as osteocytogenesis. Previous findings indicated that hypoxia facilitates osteocytogenesis *in vitro* by regulating hypoxia-related proteins (e.g., ORP150 and HIF-1) to induce osteocyte-specific markers (*Dmp1*, *Mepe*, *Fgf23*, *Cx43*) 35. Fluid-flow shear stress is also a vital trigger factor of the number and length of dendrites relate to the dynamic changes of cytoskeleton induced by E11 11. Recently, some efforts determine some exogenous molecules with a similar capacity. It has been reported that IGF1R phosphorylate tyrosine 494 (Y494) in the cytoplasmic tail of PTH1R, which increases actin polymerization and dendrite growth, thereby promoting the transition from osteoblasts to osteocytes 36. FGF2 induces an osteocyte-like phenotype in osteogenic cells by regulating the critical gene expression of osteocytes (*Dmp1*, *E11*, *Cx43*) 37, 38. Our previous studies showed that TGF-beta strongly lengthened the cell processes, enhanced migration ability, and formed gap junctions in osteocytes 39, 40. Moreover, we have demonstrated that FGF7 treatment modulated osteocyte cell processes in MLO-Y4 cell line by upregulating Wnt beta-catenin signaling pathway 9. FGF ligands interact with their signaling FGFRs which are regulated by the extracellular environment, through proteoglycan cofactors and extracellular binding proteins. Downstream of the activated signaling tyrosine kinase FGFRs, intracellular signaling cascades are also tightly associated with cytosolic adaptor proteins and the Ras-MAPK, PI3K-AKT, PLCγ, and STAT intracellular signaling pathways 1, 2. Although significant advances have been made in FGF-mediated signaling pathways, there are relatively few studies devoted to FGF7 signaling, especially in the skeletal system. FGF7 rapidly activates ERK1/2 signaling in human endometrial carcinoma cells. In contrast to the activation of MAPK pathway, FGF7 has no effect on the activation of phosphorylated PKC, phosphorylated Akt, and phosphorylated STAT3 41. The a-subunits of the G proteins (Gai1/3) are located downstream of KGFR and upstream of growth factor receptor binding 2-associated binding protein 1 (Gab1) and are required for FGF7-induced PI3K-AKT-mTORC1 activation in mouse embryonic fibroblasts (MEFs) and primary keratinocytes 42. The multiple or even opposite biological functions of FGF7 are the results of a fine balance and cross-talk between different signaling cascades activated by the growth factor. In most epithelial cells, FGF7 signaling leads to proliferation and migration, while in other cell types, it can induce cell cycle arrest and/or differentiation 43. Therefore, the activation of different biological functions by FGF7 depends on the cellular type and cellular environment.

In summary, the present study first demonstrated that FGF7 enhanced the expression of E11. Osteoblasts treated with FGF7 extended more and longer cellular processes with adjacent cells, which further promoted gap junctions formation and cell-cell communication. Consistent with our previous work, FGF7 also increased the expression of Cx43, which plays an essential role in the formation of functional gap junctions in osteoblasts. Besides, Cx43 and E11 interact directly at the cell-cell contact. Finally, we showed that the MAPK pathway and the PI3K-AKT pathway were involved in the regulation of E11 and Cx43. Therefore, our data demonstrated the role of E11 in FGF7-mediated cell-cell communication in osteoblasts.

We admit some limitations of this study. As far as we know, dendritic osteocytes form fine-tuned cellular networks and communicate with neighboring cells through gap junctions, cadherins, and other molecular structures at the sites of cell-cell contact. We observed an increase in the number and length of cell dendrites in response to FGF7, and we demonstrated that E11 and Cx43 were partly involved in this process. However, other important factors impacting the cell-cell contact and the dendrites elongation remain to be further explored. Some studies showed that the transition from osteoblasts to osteocytes also consistents with up-regulating osteocyte markers such as *Dmp1*, *Phex*, *Mepe, Sost,* and down-regulating osteoblast markers such as *Col-I*, *Bglap*, and *Alpl*. However, the mechanisms behind osteocytogenesis remain unclear. Whether FGF7 is an upstream molecule remained to be further studied. Since E11/Podoplanin is a transmembrane glycoprotein but lacks any apparent enzymatic motif with its structure, it needs to find its binding partners and work through protein-protein interactions. Previous studies have identified CD44 and ERM (ezrin and moesin) directly interact with E11 and are associated with increased directional cell migration and epithelial-mesenchymal transition 44, 45. However, specific binding sites and functions of the interaction between E11 and Cx43 remain further studied.

## Supplementary Material

Supplementary figures.

## Figures and Tables

**Figure 8:**
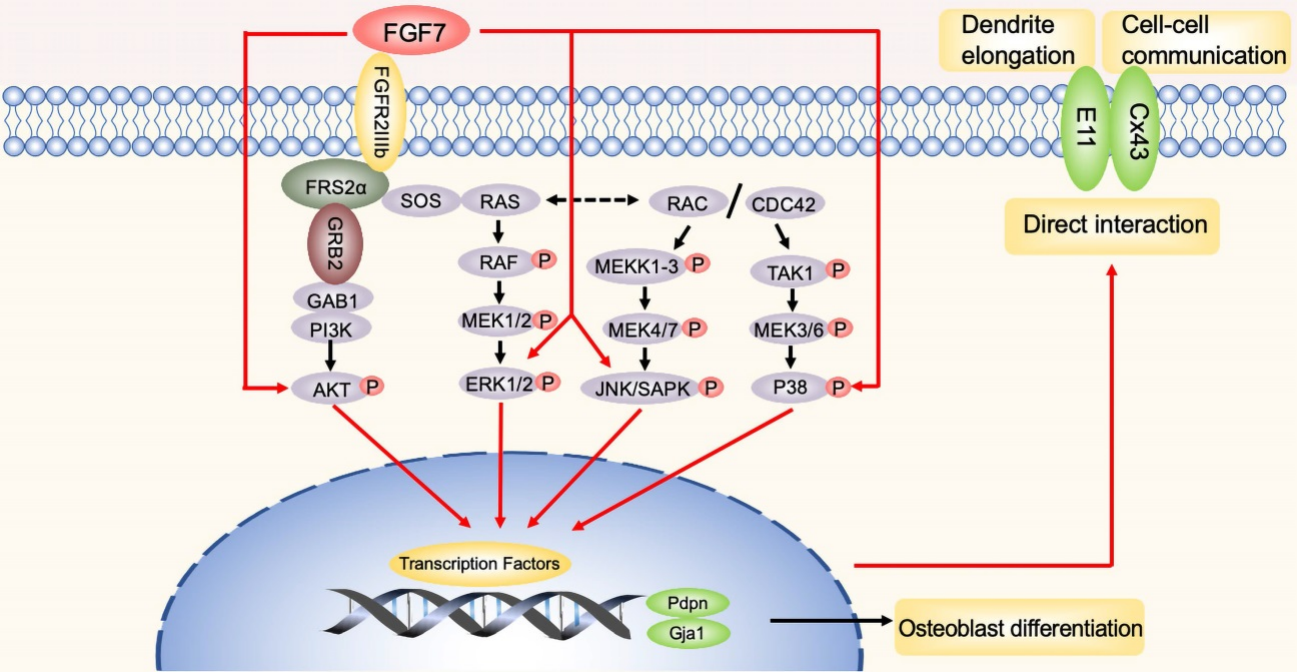
** The schematic diagram illustrates the biomechanism.** In brief, red arrows represent FGF7 induced the MAPK pathway and PI3K/AKT pathway to promote E11 and Cx43, thus facilitating dendrites elongation and cell-cell communication observed during the study. Furthermore, E11 has a direct interaction with Cx43 in osteoblasts. The black arrows represent the potential involvement of the signaling pathways that were not shown in this study.

## Abbreviations

FGF: fibroblast growth factor
FGFR: fibroblast growth factor receptor
BMSCs: bone marrow stromal cells
MSCs: mesenchymal stem cells
E11: podoplanin
Cx43: connexin43
FITC: fluorescein isothiocyanate
DAPI: 4'6-diamidino-2-phenylindole
CLSM: confocal laser scanning microscope
SL/DT assay: scrape loading and dye transfer assay
GJIC: gap junction intercellular communication
