# Supplementary material for: FGF7-induced E11 facilitates cell-cell communication through connexin43
Source: Int J Biol Sci. 2021 Sep 3;17(14):3862–74. doi: 10.7150/ijbs.65240 (PMC8495393; doi:10.7150/ijbs.65240)
Supplement: Supplementary file 1 — Supplementary figures. [file ijbsv17p3862s1.pdf]

Supplementary figures for original article:

**FGF7-induced E11 facilitates cell-cell communication through connexin43**

**Supplementary figures**

**Figure S1**

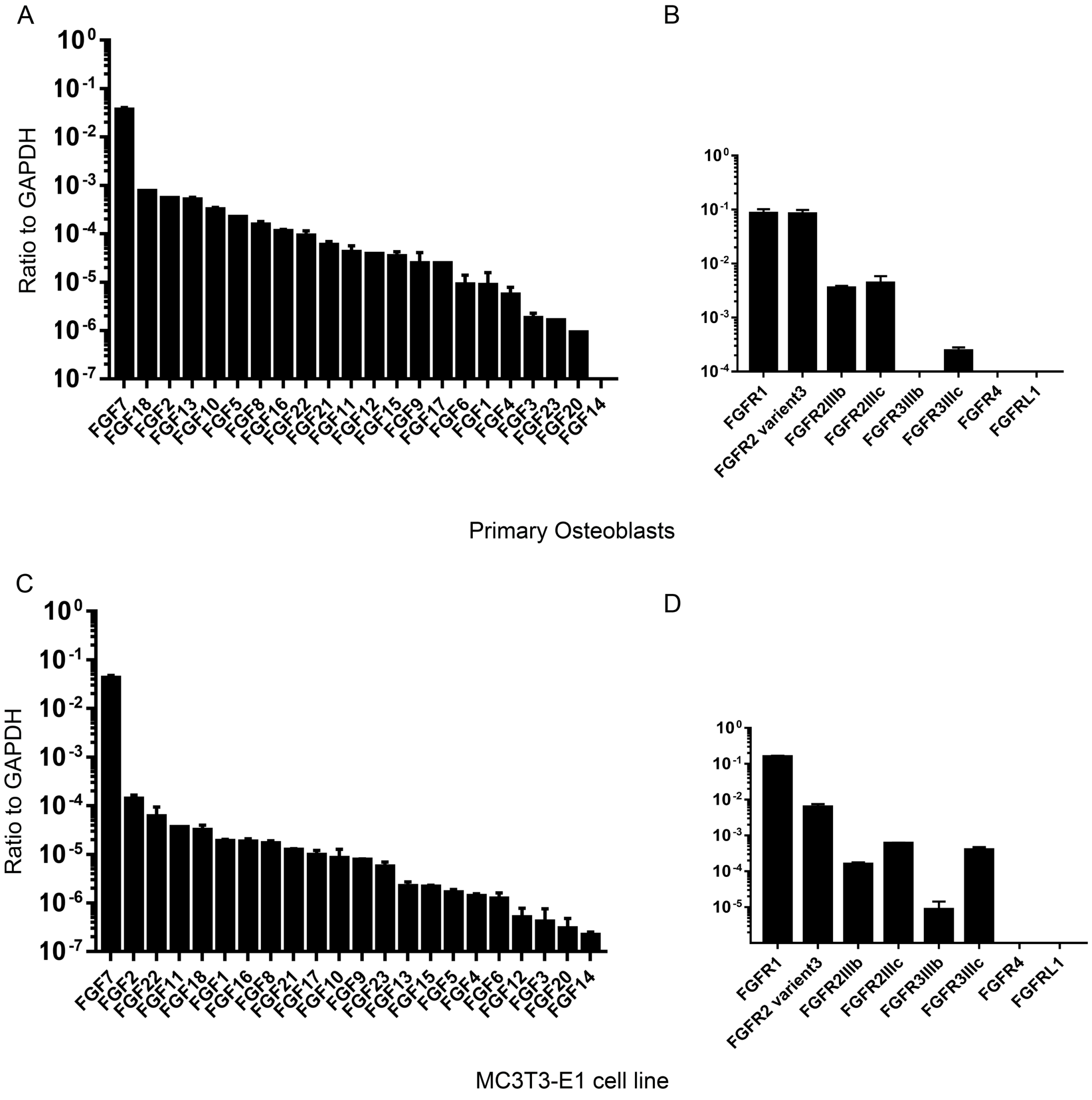


**Figure S1. The high expression of *Fgf7* in primary osteoblasts and MC3T3-E1 cell line.**

(A). qPCR results showing the expression of the whole fibroblast growth family members in primary osteoblasts and found that the expression of *Fgf7* was higher than other members.

(B). qPCR results showing the expression of the subtypes of fibroblast growth family receptor members in primary osteoblasts.

(C). qPCR results showing the expression of the whole fibroblast growth family members in the MC3T3-E1 cell line and found that the expression of *Fgf7* was higher than other members.

(D). qPCR results showing the expression of the subtypes of fibroblast growth family receptor members in MC3T3-E1 cell line.

**Figure S2**

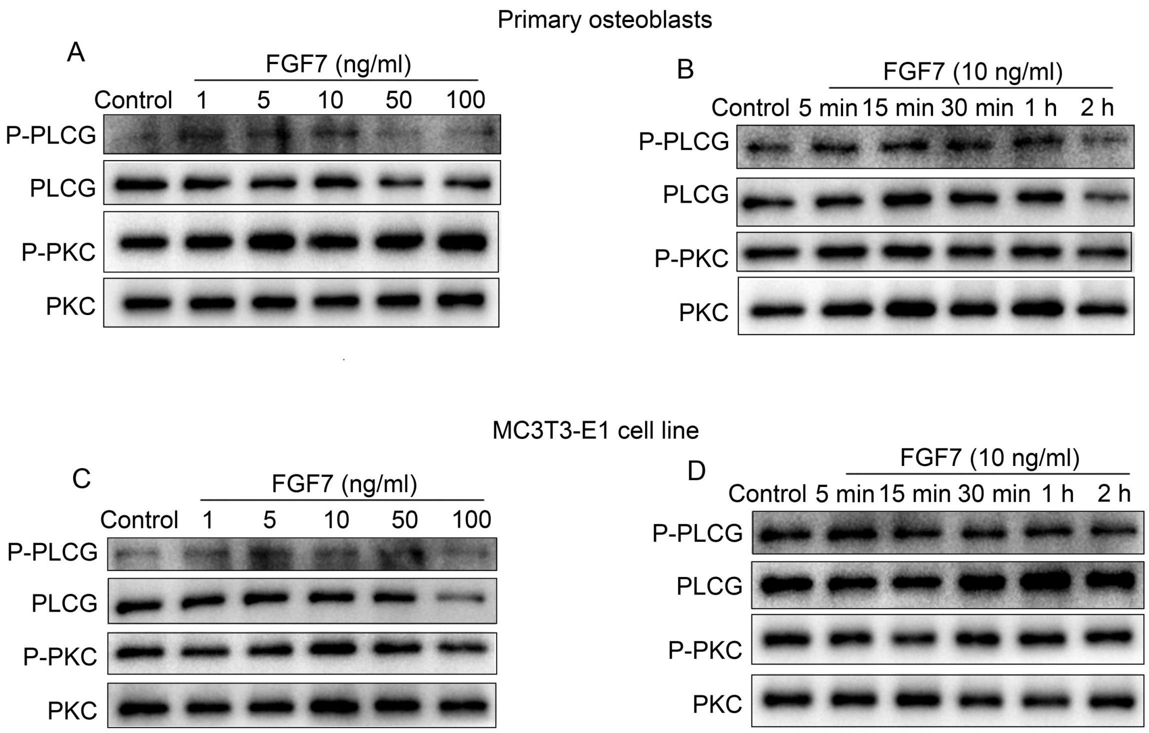


**Figure S2. Effect of FGF7 on PLC/PKC pathway activation.**

(A-D). Western blotting showing P-PLCG/PLCG and P-PKC/PKC ratios did not increase in both concentration and time-dependent manner in primary osteoblasts and MC3T3-E1 cell line in response to FGF7. The results were based on three independent experiments (n = 3).
